# Accelerated nucleophilic substitution reactions of dansyl chloride with aniline under ambient conditions via dual-tip reactive paper spray

**DOI:** 10.1038/s41598-020-78133-4

**Published:** 2020-12-09

**Authors:** Norfatirah Muhamad Sarih, David Romero-Perez, Behnam Bastani, Monrawat Rauytanapanit, Cedric Boisdon, Thanit Praneenararat, Hairul Anuar Tajuddin, Zanariah Abdullah, Abraham K. Badu-Tawiah, Simon Maher

**Affiliations:** 1 Department of Electrical Engineering and Electronics, University of Liverpool, Brownlow Hill, Liverpool, L69 3GJ UK; 2 Department of Chemistry, Faculty of Science, University of Malaya, 50603 Kuala Lumpur, Malaysia; 3 Department of Chemistry, Faculty of Science, Chulalongkorn University, Phayathai Rd, Pathumwan, Bangkok, 10330 Thailand; 4 Department of Chemistry & Biochemistry, Ohio State University, Columbus, USA

**Keywords:** Mass spectrometry, Analytical chemistry, Chemical synthesis, Mass spectrometry

## Abstract

Paper spray ionization (PSI) mass spectrometry (MS) is an emerging tool for ambient reaction monitoring via microdroplet reaction acceleration. PSI-MS was used to accelerate and monitor the time course of the reaction of dansyl chloride with aniline, in acetonitrile, to produce dansyl aniline. Three distinct PSI arrangements were explored in this study representing alternative approaches for sample loading and interaction; conventional single tip as well as two novel setups, a dual-tip and a co-axial arrangement were designed so as to limit any on-paper interaction between reagents. The effect on product abundance was investigated using these different paper configurations as it relates to the time course and distance of microdroplet travel. It was observed that product yield increases at a given distance and then decreases thereafter for all PSI configurations. The fluorescent property of the product (dansyl aniline) was used to visually inspect the reaction progress on the paper substrate during the spraying process. Amongst the variety of sample loading methods the novel dual-tip arrangement showed an increased product yield and microdroplet density, whilst avoiding any on-paper interaction between the reagents.

## Introduction

It is known that chemical synthesis can be achieved using charged microdroplets^1^ whereby the product(s) can be collected by spraying a reaction mixture toward a target/collector surface under ambient conditions^2,3^. Moreover, the reaction mixture can be monitored online by spraying directly into a mass spectrometer for analysis^4,5^. Amongst the plethora of available ionization methods, there is a distinct class known as ambient ionization (AI) that specifically refers to ionization conducted within the ambient environment, outside of the mass spectrometer, and with minimal sample preparation^4,6–11^. Spray-based AI methods create a localized environment, within charged microdroplets, that is conducive for reactions to occur between constituents of the solution^12–14^. Paper spray (PS), also known as paper spray ionization (PSI), sits firmly in the ambient ionization family^15–17^. PS is a popular AI technique as it can enable effective ionization and transport of charged droplets in the open air environment external to the mass spectrometer, with no pneumatic assistance needed as well as minimal requirement for prior sample preparation^18–20^. AI techniques can, in most cases, be classified into three major categories: liquid extraction, laser ablation and plasma desorption^21,22^. Within the category of liquid extraction, PSI can be further considered a substrate spray technique since ionization is performed directly from the substrate where the sample is located^23^. A number of studies have been carried out investigating and optimising the key parameters that influence the analytical performance of PS^24–26^. In a conventional sense, PS-mass spectrometry (MS) has been widely utilized for many types of sample analysis including environmental^27–29^, biological^30–34^, therapeutics^35–39^, drugs^40–43^ and chemical warfare agents^44,45^.

In addition to its primary application for the purpose of analysis, PS-MS is an emerging tool for rapid reaction monitoring as it can be used in a ‘reactive mode’ to perform organic reactions while generating molecular ions under ambient conditions^18,28,46–49^. In a typical PS experiment, reagents are loaded onto a triangular paper surface by pipetting (i.e., drop-casting). Once a sufficient electrical potential is applied with an accompanying spray solvent, the analyte(s) is transferred from the paper in the form of electrosprayed microdroplets^50,51^, which can then be directly characterized by MS. It is thought that either the liquid thin film on the paper substrate or the charged microdroplets, or both, are the means for the accelerated reaction, and the contributions from each depends on the degree of solvent evaporation at each location^47,52,53^. In the PS experiment, reactions are carried out by using small amounts of reagents with subsequent mass spectrometric assessment providing insight regarding certain aspects of the chemical reactivity. Recently, several classical reactions have been carried out using reactive AI-based methods, including: Katritzky reaction^18^, Claisen–Schmidt base-catalyzed condensations^4^, haloform reactions^46^ and synthesis of carboxylic acids from alcohols^50^.

**Scheme 1:**
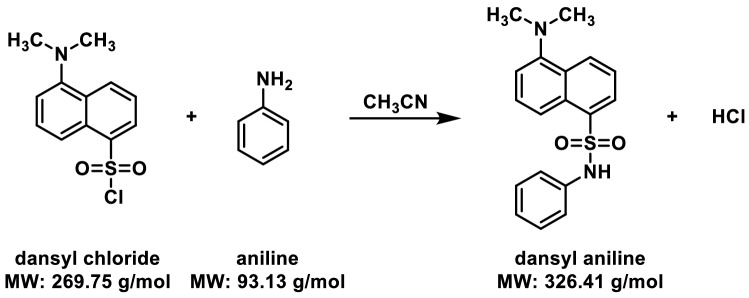
The synthesis of dansyl aniline.

**Figure 1:**
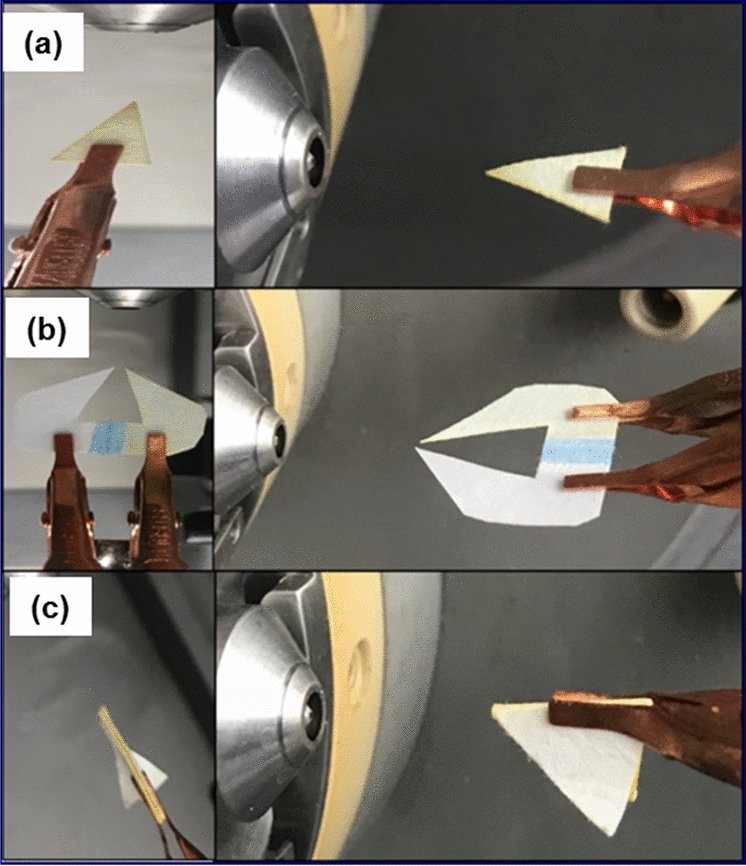
Photograph of the three PS arrangements: (**a**) single tip, (**b**) dual-tip, and (**c**) co-axial.

Dansyl derivatives are known for their fluorophoric characteristics, with good photo-physical properties, including a large Stokes’ shift and absorbance in the near UV region^54,55^. One such example is dansyl aniline, a fluorescently labelled aniline that is used in the manufacturing of dyes, medicines, resins, varnishes, perfumes, vulcanizing rubber and even as a solvent. Recently we combined a simple mixture of fluorescent organic compounds including furocoumarin^56^ and dansyl aniline, to produce almost pure white light emission (WLE) from a narrow band UV LED^57^. The fluorescent property of dansyl aniline also enables us to monitor various aspects of the reaction optically (before, during and after the reaction) on both the paper spray emission substrate and the target/collector substrate. One of the beauties of ambient ionization is that it takes place outside of the MS instrument and so enables reaction products to be characterized and monitored by other analytical techniques. In this study, we examine the time course of the reaction for the nucleophilic substitution reaction of dansyl chloride with aniline to yield dansyl aniline (Scheme 1). This was investigated with PSI-MS and compared with the bulk solution-phase reaction, as monitored by electrospray ionization (ESI)-MS. PSI-MS is usually performed in a standard configuration, with a single macroscopic triangular tip (isosceles triangle) cut from chromatography paper^18^. Here, we explore three different substrate configurations, including: a traditional single tip and two novel arrangements: dual-tip and a co-axial paper setup (Fig. 1), as representing three types of sample loading and interaction. The idea regarding the two proposed novel arrangements is to limit (or entirely negate) the solution phase (i.e., on-paper) interactions between the reagents (Fig. S1). The effect on product ion abundance was investigated from the different types of paper tip configuration by considering the distance of droplet travel and droplet distribution.

## Results and discussion

**Figure 2:**
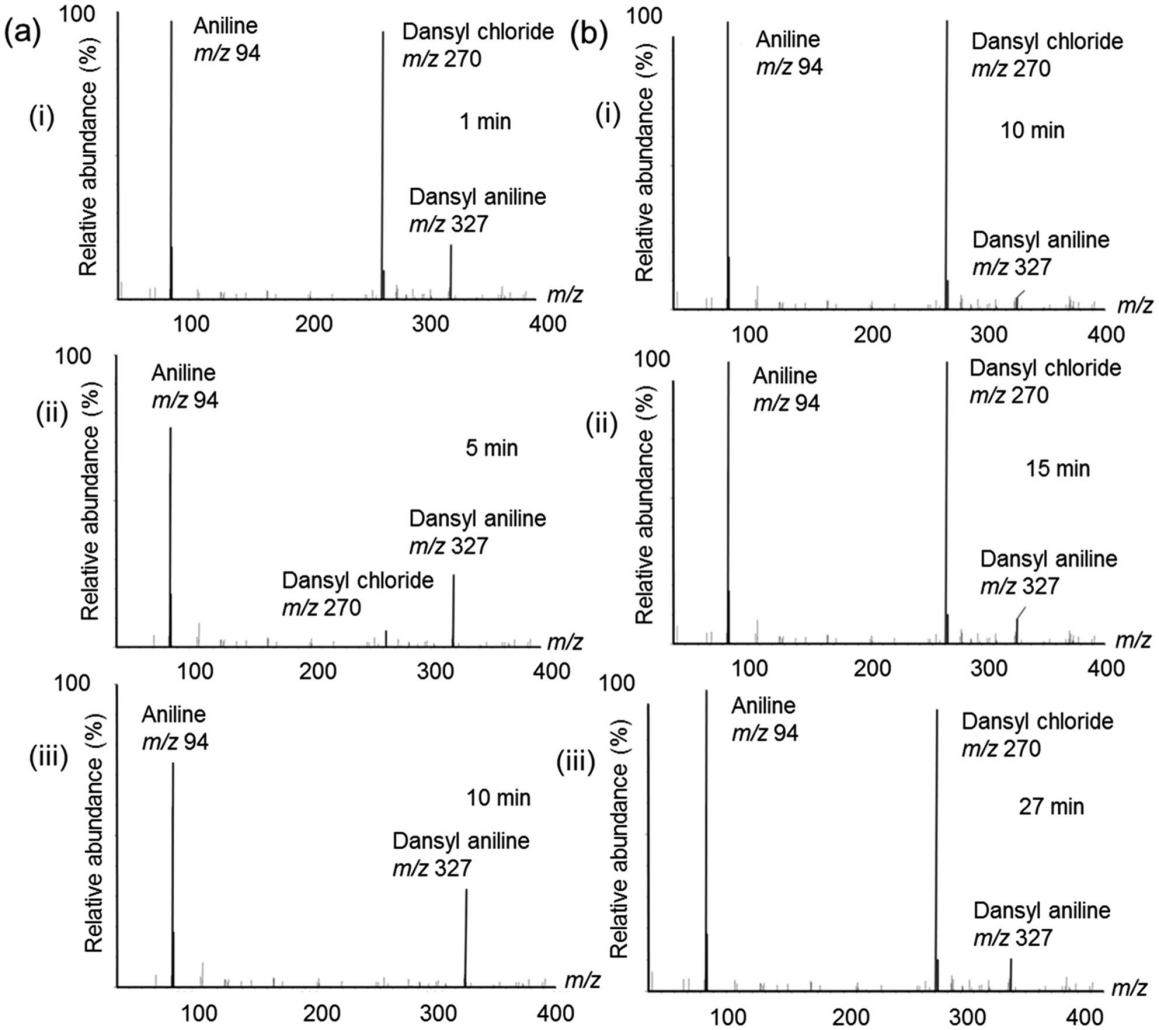
Time-resolved mass spectra showing the synthesis of dansyl aniline (protonated molecular product ion indicated at *m*/*z* 327). Unreacted aniline and dansyl chloride are evident from their respective mass spectral peaks: [aniline + H]^+^ at *m*/*z* 94 and [dansyl chloride + H]^+^ at *m*/*z* 270. Three distinct time intervals are given, as indicated by (i)–(iii) which are given in the subfigures for both: (**a**) single tip (conventional) PSI-MS and (**b**) ESI–MS.

A nucleophilic substitution reaction between dansyl chloride and aniline, conducted under reactive paper spray conditions, yielded dansyl aniline as expected. ESI–MS was also used to analyze a mixture of dansyl chloride and aniline at the same concentration in acetonitrile solution, acting as a control experiment. Mass spectra of the product ions, as well as reactant ions, recorded as a function of the dwell time of the solution on the single tip paper substrate, are shown in Fig. 2a. Here, the reagents (equimolar mixture, 100 μM each) were pre-mixed before drop-casting onto the paper triangle (time of mixing before drop cast was less than 1 min). The aniline reagent (*MW* 93) reacts with dansyl chloride (*MW* 269) to yield the corresponding dansyl aniline product detected at *m/z* 327. The unreacted reagents, aniline and dansyl chloride were also detected at *m/z* 94 and *m/z* 270 as protonated species, respectively. Product formation was fast with the dansyl chloride peak at *m/z* 270 disappearing in just 10 min dwell time (Fig. 2a,iii). In comparison with the bulk solution-phase reaction from the control experiment, using the same equimolar concentration of reagents (see ‘Materials and Methods’), a high abundance of unreacted dansyl chloride was observed, even after 27 min of reaction time (Fig. 2b). Given that equal amounts of aniline were used in both reaction conditions, the relative ion intensities provide a reasonable indicator for monitoring reaction progress. In general, reaction yields from online MS can act as a rough estimate for product amount and conversion rates were calculated at each time point as per Fig. 2 (Supporting Information, Table S1)^12^. Further inspection reveals that the 27 min bulk-phase reaction shows a strong resemblance with the on-paper reaction after only 1 min (i.e., Fig. 2a,i versus Fig. 2b,iii). An enhancement factor more than ~ 20 times in favor of the paper-based reaction can be estimated assuming comparable product yield for both reaction conditions. Compared to the control experiment, only one reagent (dansyl chloride) showed 100% consumption for PSI-MS, even though equimolar amounts were used in the experiment. Since dansyl chloride is a moisture sensitive compound^58^ it is susceptible to hydrolysis by water and hydroxyl ions^59^ from the ambient environment. In order to obtain an improved product yield, excess amount of dansyl chloride would be needed for the PS experiment.

**Figure 3:**
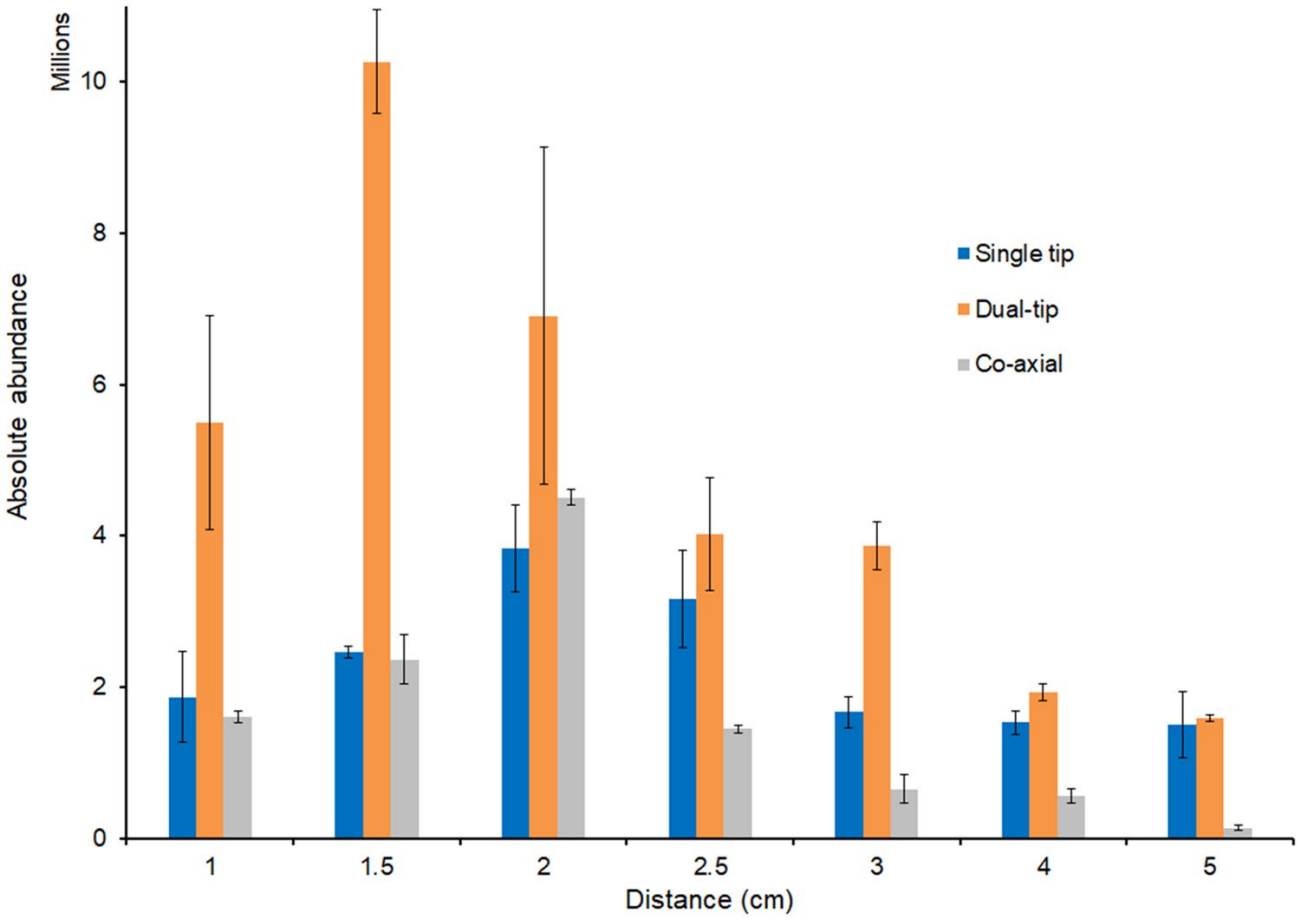
Absolute abundance of product ion (*m*/*z* 327) from each paper arrangement loaded at different distances. The error bars indicate the standard deviation from three replicates.

To further investigate this observation, we developed the co-axial and dual-tip arrangements, in order to minimize any on-paper interaction compared to a classical PS approach (i.e., using a single, macroscopic tip), so that any reactions should occur predominantly in the desolvating droplets. To shed light on this possibility, we first examined the effect of distance for the various paper configurations to explore the effect of droplet travel on the reaction progression. The distance from the paper tip to the MS inlet was systematically increased from 1 to 5 cm. As can be seen in Fig. 3, the dual-tip paper arrangement consistently gave the highest product ion yield. The rise and then fall of the product signal confirms the absence of any major effect on product formation, except the distance of droplet travel on the timescale of the experiment. From Fig. 3, for all paper spray configurations, the initial rise in product ion signal with increasing distance is attributed to solvent evaporation effects, while the decrease in signal (after ~ 1.5 to 2 cm distance) is attributed to reduced ion sampling efficiency at the atmospheric pressure interface of the mass spectrometer. The decrease in droplet size caused by solvent evaporation over longer distances increases the reaction rate by concentrating charged species at the surface, albeit at the expense of some signal loss (due to spray divergence)^12,47,60–62^. Bain et al. suggest that the faster reaction rate of molecules at surfaces is a result of the partial desolvated nature of surface-active species contributing to reaction acceleration^63^. The fact that the dual-tip consistently gave the highest product ion abundance at each distance investigated is attributed to effective reagent mixing at the Taylor cone, followed by reaction acceleration in the droplet-phase where solvent evaporation is more pronounced. Whilst we cannot rule out gas-phase reactions, we expect contributions to be insignificant due to high diffusion rates of gas-phase reactants with limited confinement under atmospheric conditions^64^. The vapor pressure of aniline is 0.04 kPa at 20 °C^65^ and that of dansyl chloride is expected to be negligible at room temperature^66^, which further suggests that gas-phase contributions should be relatively small.

**Figure 4:**
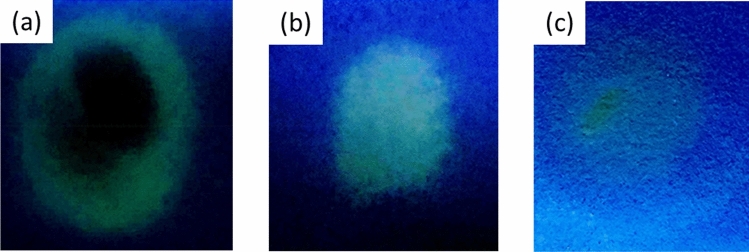
Image of paper substrate collection surface (i.e., target) whereby 0.1 M of dansyl chloride and 0.1 M aniline solution were sprayed from: (**a**) single tip, (**b**) dual-tip and (**c**) co-axial tip arrangements at a distance of 2 cm (as labelled in the image).

The distance effect suggests that the ambient interaction of the droplets is an important feature. In this regard the product material was collected on paper substrates from spraying the reagents for each of the paper tip arrangements (Fig. 4), which shows, qualitatively, that the dual-tip gives the brightest yellow emission light (indicating dansyl aniline formation). Another observation is that the conventional single tip leads to the majority of product being formed in an outer ring (Fig. 4a). We suspect that this is due to the fact that smaller droplets are more likely to be found in the outer regions of the Taylor cone formation, as is common for electrospray-type events^67^. Hence, the product is more likely to form through reactions where the smaller droplets are present leading to the outer ring of increased fluorescence intensity. Interestingly, for the dual-tip arrangement (Fig. 4b) the fluorescent product is located predominantly at the centre of the target collection surface, where we suspect the individual plumes from the two spray tips interact. This was confirmed by imaging the dynamic spray process under laser light illumination (see supporting information, Fig. S2) where it is clear that two distinct spray plumes overlap with each other.

**Figure 5:**
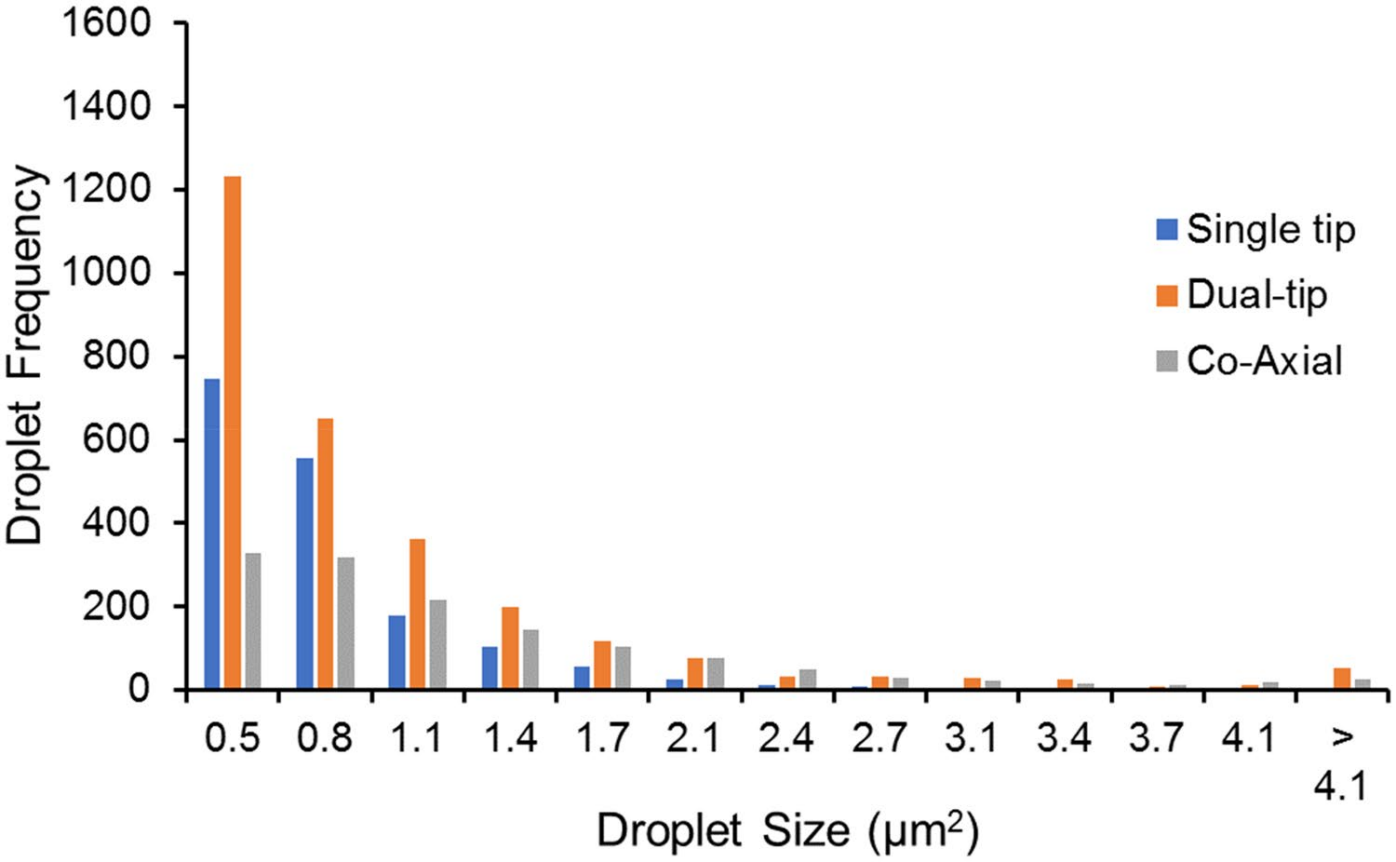
Droplet distribution for the three paper loading types. Each was sprayed on to an ITO coated glass slide at a distance of 5 mm and the central region of the slide was examined in each case to obtain the droplet distribution (see ‘Materials and Methods’).

Figure 5 shows the droplet size distribution for each arrangement. Droplet size information was generated by pulsing the voltage to each PS arrangement, spraying towards a conductive glass slide and then afterwards imaging the resultant droplets deposited on the slide (see ‘Materials and Methods’). The dual-tip paper produces the highest frequency of the smallest droplets that can be imaged with the setup. Moreover, the dual-tip arrangement produced the most droplets overall and the largest droplet density (see supporting information, Figs. S3, S4).

**Figure 6:**
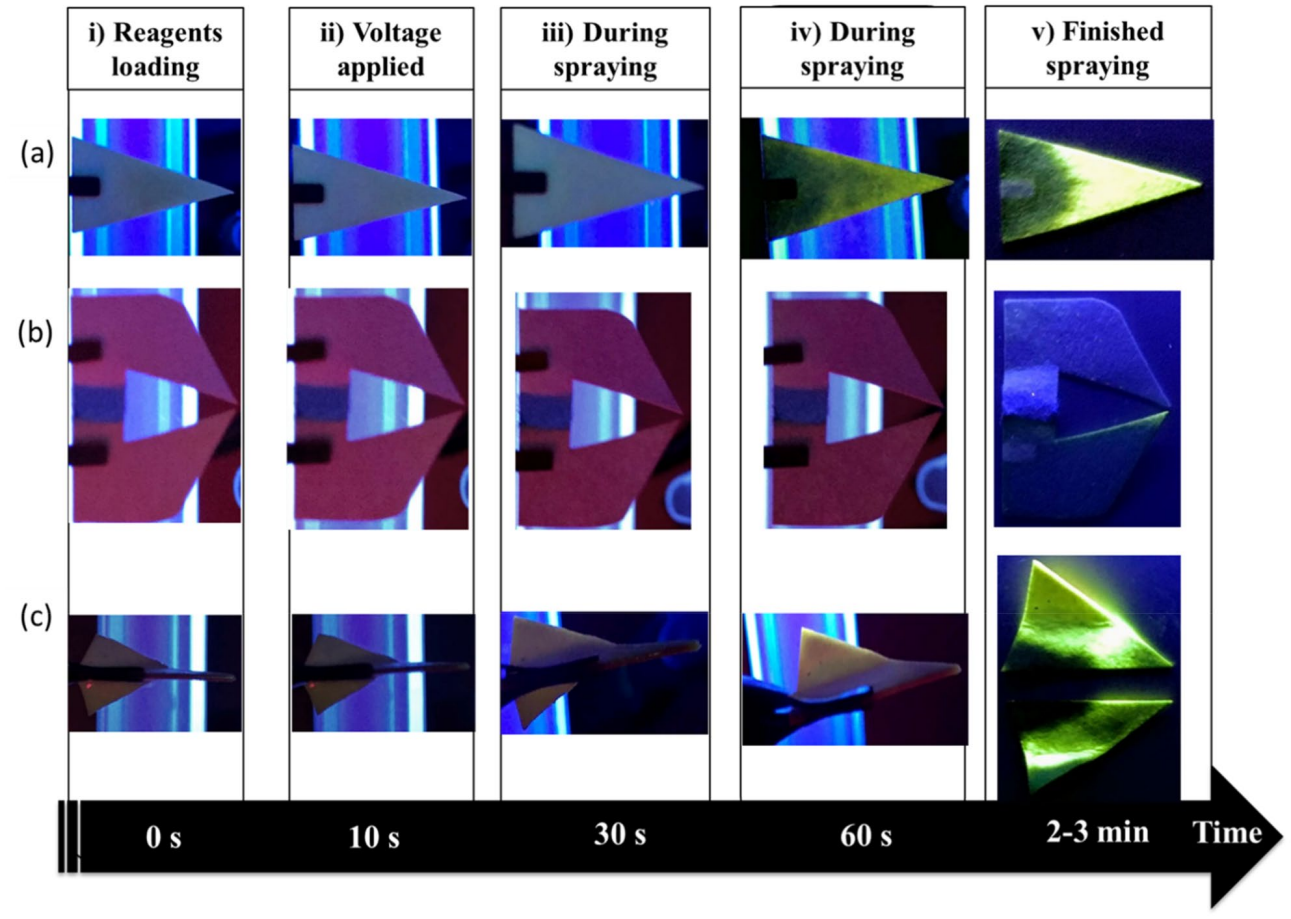
Flow of spraying observation over time, under UV light from each of the paper tips: (**a**) single tip, (**b**) dual-tip and (**c**) co-axial.

In the dual-tip arrangement, the two reactants are spotted on either side of the paper substrate as separated by a hydrophobic (wax) barrier in order to isolate the two regions (see wax barrier, blue color, in Fig. 1b). This significantly inhibits the possibility of any contact on the paper and thus any interaction between the two reactants is predominantly in the microdroplet/gas phase. To confirm this, the reaction progression was also examined under UV-light illumination for each of the paper tips. Still images were captured at distinct instances: i) reagents loading, ii) when high voltage is first applied, iii) after 30 s of spraying, iv) after 1 min of spraying and v) after spraying had completed (Fig. 6). It is shown that the fluorescence of dansyl aniline (a yellow emission light) starts to appear for single tip and co-axial on the paper substrate during spraying but not for the dual-tip arrangement. The coaxial arrangement consists of two paper triangles, each with one of its sides sealed with wax. These two papers are held together by the hydrophobic waxed side yet there is evidence of some reagent leakage and interaction as can be seen in Fig. 6c; note in Fig. 6cv) after spraying has finished, the ‘wings’ of the co-axial arrangement have been opened up (i.e., unfolded). However for the dual-tip arrangement, there is virtually no interaction of reagents on the paper and therefore no on-surface product formation. This confirms reaction products detected by MS are formed exclusively in the charged microdroplets (or gas phase). This observation can have profound implication for the mechanism of reaction acceleration in desorption electrospray ionization^68^ and contained-electrospray ionization^69^, in which the co-existence of both liquid thin film and droplets makes it difficult to discern the specific microreactor system responsible for reaction acceleration. In the current case, the dual-tip paper allows us to decouple the influence of liquid thin film from that of charged droplets in paper spray. By comparing the droplet-only dual tip to the conventional paper tip, where both thin film and droplets are involved in the reaction, we show for the first time that charged droplets can be a more reactive pathway.

## Conclusions

Accelerated nucleophilic substitution reactions of dansyl chloride with aniline under ambient conditions via reactive paper spray were successfully carried out. Three PS tip configurations were investigated, and it is shown that a novel dual-tip arrangement leads to an improvement in product ion yield due to rapid solvent evaporation in the charged microdroplet environment. Since dansyl aniline is a fluorophore, it allows optical observations to be made throughout the PS reaction. This confirmed that for the dual-tip arrangement there is no interaction between the reagents on the paper. However, the co-axial setup, also designed for this purpose, showed that it did not work as intended and the approach can be improved in future work to ensure that the hydrophobic barrier is upheld. For the dual-tip setup it was observed that product formation occurs almost exclusively in the microdroplets, in the ambient air, after the reagents have left the paper substrate. This result provides further support to recent phenomenon involving accelerated droplet reactions. The dual-tip paper spray arrangement for reactions is different from both ESI and desorption electrospray ionization (DESI) in that no reagent pre-mixing or liquid thin film formation are involved, making it straightforward to confirm that reactions were accelerated solely in the droplets during flight toward the mass spectrometer. The droplet distribution from the different paper spray configurations was examined and showed that the dual-tip arrangement had the largest droplet density. Due to its simplicity, ease of implementation and speed of analysis, this approach shows promise as a fast and insightful tool for preliminary reaction screening, reaction monitoring and as a means to explore mechanistic considerations.

## Materials and methods

All reagents and solvents were of analytical grade or higher and were used directly without further purification. Dansyl chloride, aniline and HPLC-grade acetonitrile were purchased from Sigma–Aldrich (UK). The filter paper used for paper spray ionization was Whatman grade I, from Whatman International Ltd (Maidstone, UK).

### The paper spray tip configurations

For PS-MS, three different PS tip configurations were tested to investigate the influence of sample loading. This included a typical single PS isosceles triangle (Fig. 1a) with the following dimensions, base: 5 mm, height (from base to tip): 8 mm. The dual-tip arrangement is one single substrate which essentially consists of two triangular apexes in close proximity (Fig. 1b). The same high voltage is supplied to both regions simultaneously which are separated by a hydrophobic wax barrier (shown as blue line on the dual-tip). The co-axial configuration consists of two triangular papers each with a wax coating^70^ on one side that are folded and clipped together (Fig. 1c). The dimensions of the three different paper spray configurations are illustrated as in Fig. S1 and have been explained further in the supporting information. The idea regarding the two proposed novel arrangements is to limit (or entirely negate) the solution phase (i.e., on paper) interactions between the reagents. For the dual-tip configuration, each reagent was drop-cast separately on either side of the dividing wax barrier near the apex of each tip. In a similar manner, for the co-axial setup, each reagent was drop cast on to a separate ‘wing’. Whereas, for the conventional PS arrangement, drop casting of reagents was performed at the same location near to the apex of the singular tip.

### Mass Spectrometer Settings

All PS-MS and ESI experiments were performed on a Waters Xevo triple quadrupole mass spectrometer (TQ MS). Data acquisition was accomplished with MassLynx V4.1 software. The temperature of the source block was set at 100 °C. The cone voltage was set at 20 V. No further optimizations were performed, and the remaining instrument settings were left as per the manufacturer recommendations.

### The procedure for the dansyl aniline synthesis by ESI–MS

For ESI analysis, 10 μL of 100 μM dansyl chloride and 10 μL of 100 μM aniline (in acetonitrile) were mixed together in a syringe vessel before ESI–MS analysis using a Waters Xevo TQ-MS (Waters, Wilmslow, UK).

### The procedure for the dansyl aniline synthesis under reactive paper spray conditions

The reagents were drop-cast onto paper: 5 μL of 100 μM dansyl chloride and 5 μL of 100 μM aniline (in acetonitrile). The experiment was carried out and compared for each of the paper tip configurations, whilst varying the spray distance (1, 1.5, 2, 2.5, 3, 4 & 5 cm) from the apex of the tip to the source inlet of the mass spectrometer (Waters Xevo TQ-MS), with + 6 kV applied.

### Observations of reactive paper spray for the fluorescent product using UV-light

The experiment was carried out for each of the paper tips by spraying the reagents (25 μL of 0.1 μM), at ~  + 6 kV (at a distance = 2 cm) and collecting onto a paper target (acting as a collection surface). In the experiment, the paper tips were connected to a positive electrode while the paper target was placed on top of a grounded electrode. A paper target was used as a collection surface solely for the purpose of capturing the emitted droplets for subsequent visual inspection via UV light illumination. Both the paper spray and paper target substrates were observed *in-situ* under UV light illumination (at λ_ex_ = 340 nm), in a dark room. Using paper as the collection target is convenient and enables the product to be effectively captured during the experiment with minimal evaporative losses.

### Optical inspection of the paper spraying process

A decollimated green laser diode (NICHIA NUGM03T) with a peak emission at 525 nm was used to visualize the dynamic spray process. A Nikon 5300 camera with 35 mm lens and a macro add-on; exposure: 1/30 s, f/8, 35 mm, ISO-100. A lab power supply was used to provide + 6.5 kV to the Whatman grade 1 paper substrate which was placed 1 cm from a grounded target with acetonitrile used as the spray solvent.

### Characterizing the droplet distribution

**Figure 7:**
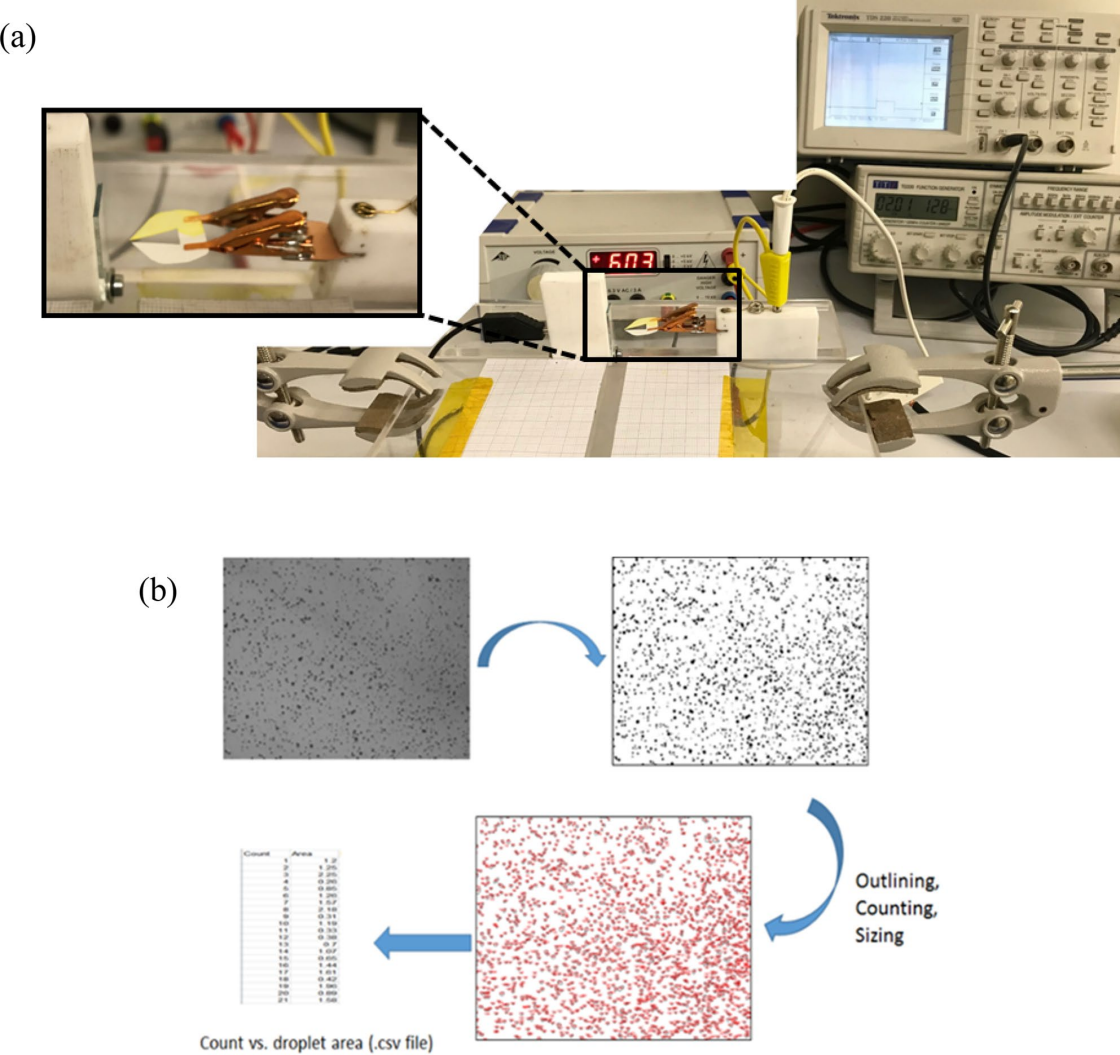
(a) Experimental set-up for the droplet distribution (e.g., dual-tip). (b) Typical work flow for the image processing steps.

The droplet distribution experiment (Fig. 7a) is carried out for each of the paper configurations by spraying onto an ITO coated glass slide using 2 pulses, each 50 ms in duration, at ~  + 6 kV (at a distance = 5 mm) for each distribution. The glass was examined under a microscope (× 40 lens) and images were captured from the central region of the glass slide. The images were then processed using ImageJ software to determine the droplet counts and distribution for each configuration (as per Fig. 7b).

## Supplementary information

Supplementary Information.
