# Supplementary material for: Accelerated nucleophilic substitution reactions of dansyl chloride with aniline under ambient conditions via dual-tip reactive paper spray
Source: Sci Rep. 2020 Dec 9;10:21504. doi: 10.1038/s41598-020-78133-4 (PMC7725966; doi:10.1038/s41598-020-78133-4)
Supplement: Supplementary file 1 — Supplementary Information. [file 41598_2020_78133_MOESM1_ESM.docx]

**Supporting Information**

**Accelerated Nucleophilic Substitution Reactions of Dansyl Chloride with Aniline under Ambient Conditions via Dual-tip Reactive Paper Spray**

Norfatirah Muhamad Sarih^1,2^, David Romero-Perez^1^, Behnam Bastani^1^, Monrawat Rauytanapanit^1,3^, Cedric Boisdon^1^, Thanit Praneenararat^3^, Hairul Anuar Tajuddin^2^, Zanariah Abdullah^2^, Abraham K. Badu-Tawiah^4+^ and Simon Maher^1*^

^1^Department of Electrical Engineering and Electronics, University of Liverpool, Brownlow Hill, Liverpool, L69 3GJ, UK

^2^Department of Chemistry, Faculty of Science, University of Malaya, 50603 Kuala Lumpur, Malaysia

^3^Department of Chemistry, Chulalongkorn University, Bangkok, Thailand

^4^Department of Chemistry & Biochemistry, Ohio State University, USA

^+^Correspondence, Email: [badu-tawiah.1@osu.edu](mailto:badu-tawiah.1@osu.edu)

*Correspondence, E-mail: [s.maher@liverpool.ac.uk](mailto:s.maher@liverpool.ac.uk)


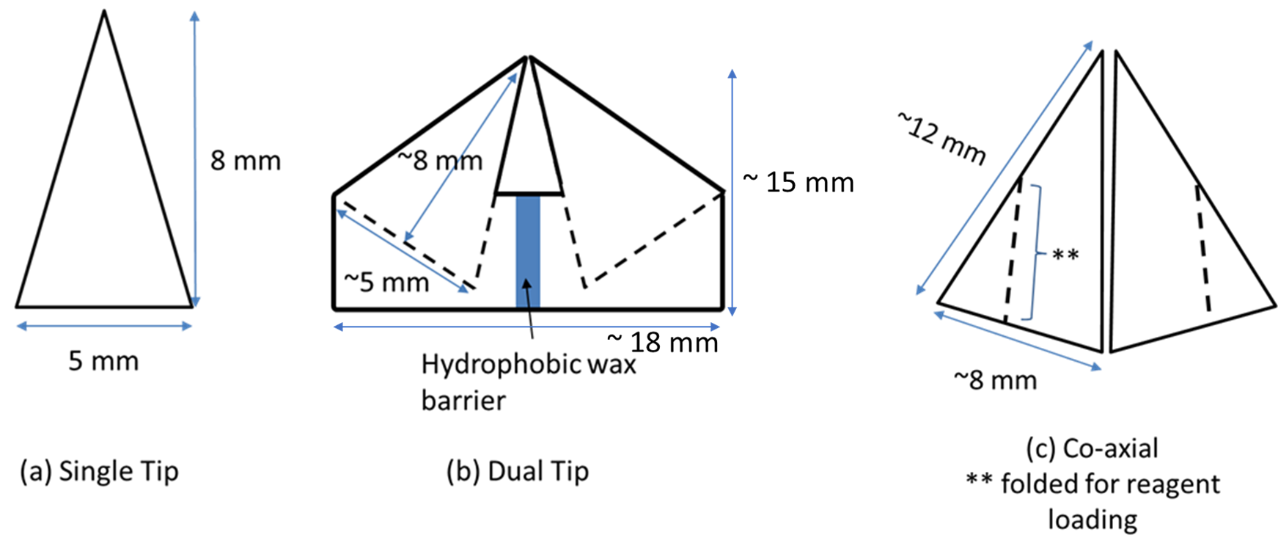


Figure S1. Dimensions of the three different paper spray configurations: (a) single tip, (b) dual-tip, (c) co-axial.

**Dual-tip configuration procedure**

A filter paper was cut into a rectangle before folding equally at the centre (i.e., where the wax barrier in indicated in Fig. S1b) and then cut sharply to form a triangle in the middle of the paper, so as to produce the two triangular paper tips from a single substrate. Next, the middle of the paper should have a line of a hydrophobic material (i.e., wax) as a barrier applied (and heated treated as necessary to ensure penetration) to separate each of the two reagents from mixing on the paper.

**Co-axial configuration procedure**

A paper substrate was printed with wax coated on one side. This substrate was then cut into two triangular papers according to the dimensions as given Fig. S1c. Then, the two triangular pieces were folded (to become as ‘wings’), as illustrated in the Fig. S1c, like a paper plane and then clipped together on their wax coated sides. The wax acts as a barrier to limit on-paper interactions between the reagents after both triangular papers were clipped together. In this case the clip also acts as the electrical connection to the paper. This can also be seen from the photograph in the main manuscript, Fig. 1c.

| **Fig. 2 Sub-Plot** | **Yield (%)** |
| --- | --- |
| 2a i PS (1 min) | 7.68 |
| 2a ii PS (5 min) | 27.32 |
| 2a iii PS (10 min) | 33.38 |
| 2b i ESI (10 min) | 1.65 |
| 2b ii ESI (15 min) | 1.80 |
| 2b iii ESI (27 min) | 2.25 |

Table S1. This data relates to Figure 2 in the main manuscript. Approximate conversion rates at each time point have been calculated according to $Yield \left( \% \right)=\left( {I_{product}}/\left( I_{product}+I_{reagent} \right) \right)\times100$ where $I_{product}$ and $I_{reagent}$ relate to the product and reagent ion peak intensities, respectively.


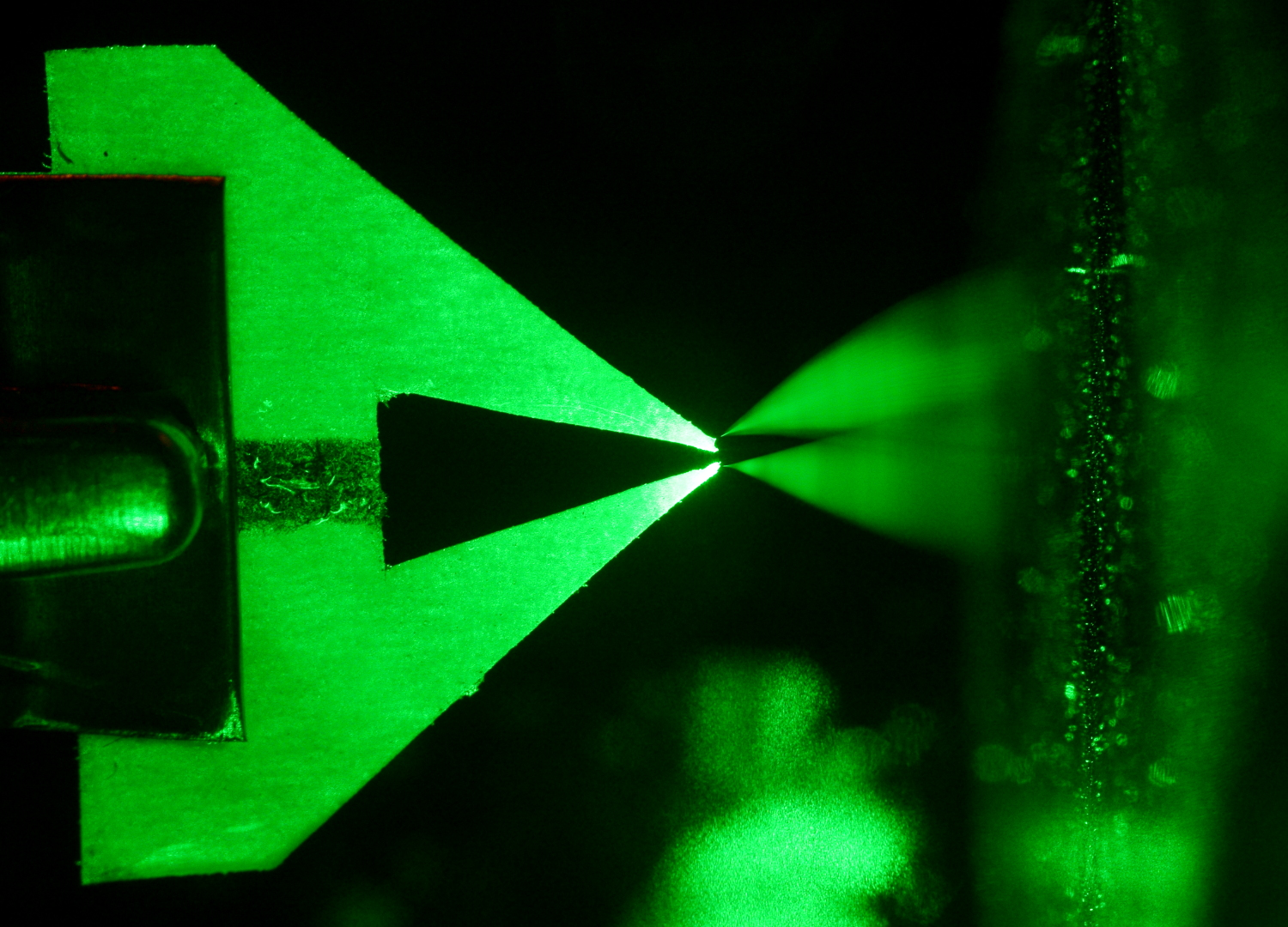


Figure S2. Photograph illustrating the dual plume genenration and interaction for the dual-tip arrangement. A decollimated green laser was used to image the dynamic spray generation. Acetonitrile is used as the spray solvent. The paper is connected to a positive voltage of +6.5 kV and placed ~1 cm from a grounded target.


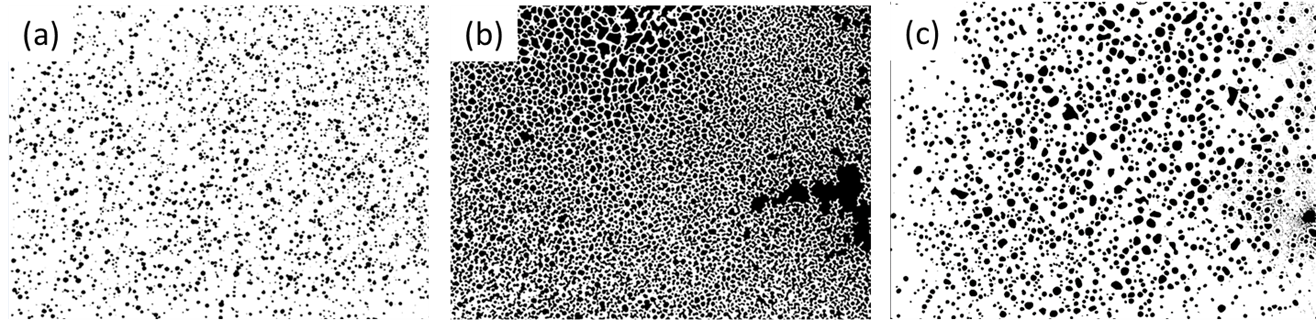


Figure S3. Droplet distribution images by spraying 0.001 mM of dansyl chloride and aniline solution from: (a) single tip, (b) dual-tip and (c) co-axial tip arrangements at a distance of 5 mm on to an ITO coated glass slide (as the target/collection surface). Imaging area: 18.13 mm x 13.60 mm (horizontal x vertical), with microscope magnification (x40 lens).


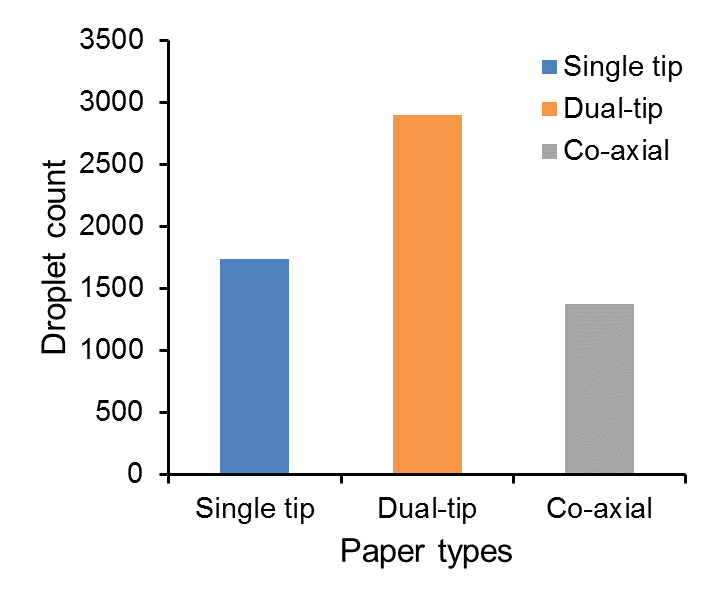


Figure S4. Total droplet count from each of the paper types (obtained from the data of figure S3).
